# White Matter Lesions and Outcomes After Endovascular Treatment for Acute Ischemic Stroke: MR CLEAN Registry Results

**DOI:** 10.1161/STROKEAHA.120.033334

**Published:** 2021-06-03

**Authors:** Simone M. Uniken Venema, Alida A. Postma, Ido R. van den Wijngaard, Jan Albert Vos, Hester F. Lingsma, Reinoud P.H. Bokkers, Jeannette Hofmeijer, Diederik W.J. Dippel, Charles B. Majoie, H. Bart van der Worp

**Affiliations:** 1 Department of Neurology and Neurosurgery, Brain Center, University Medical Center Utrecht, the Netherlands (S.M.U.V., H.B.v.d.W.).; 2 Department of Radiology, Maastricht University Medical Center Plus, the Netherlands (A.A.P.).; 3 School for Mental Health and Sciences, University of Maastricht, the Netherlands (A.A.P.).; 4 Department of Neurology, Haaglanden Medical Center, the Hague, the Netherlands (I.R.v.d.W.).; 5 Department of Neurology, Leiden University Medical Center, the Netherlands (I.R.v.d.W.).; 6 Department of Radiology, St. Antonius Hospital, Nieuwegein, the Netherlands (J.A.V.).; 7 Department of Public Health (H.F.L.), Erasmus MC, University Medical Center, Rotterdam, the Netherlands.; 8 Department of Neurology (D.W.J.D.), Erasmus MC, University Medical Center, Rotterdam, the Netherlands.; 9 Department of Radiology, Medical Imaging Center, University Medical Center Groningen, the Netherlands (R.P.H.B.).; 10 Department of Neurology, Rijnstate, Arnhem, the Netherlands (J.H.).; 11 Department of Radiology and Nuclear Medicine, Amsterdam University Medical Center, University of Amsterdam, the Netherlands (C.B.M.).

**Keywords:** acute ischemic stroke, cerebral small vessel diseases, odds ratio, radiography, registries

## Abstract

**Background:**

Cerebral white matter lesions (WMLs) have been associated with a greater risk of poor functional outcome after ischemic stroke. We assessed the relations between WML burden and radiological and clinical outcomes in patients treated with endovascular treatment in routine practice.

**Methods:**

We analyzed data from the MR CLEAN Registry (Multicenter Randomized Controlled Trial of Endovascular Treatment for Acute Ischaemic Stroke in the Netherlands)—a prospective, multicenter, observational cohort study of patients treated with endovascular treatment in the Netherlands. WMLs were graded on baseline noncontrast computed tomography using a visual grading scale. The primary outcome was the score on the modified Rankin Scale at 90 days. Secondary outcomes included early neurological recovery, successful reperfusion (extended Thrombolysis in Cerebral Infarction ≥2b), futile recanalization (modified Rankin Scale score ≥3 despite successful reperfusion), and occurrence of symptomatic intracranial hemorrhage. We used multivariable logistic regression models to assess associations between WML severity and outcomes, taking the absence of WML on noncontrast computed tomography as the reference category.

**Results:**

Of 3180 patients included in the MR CLEAN Registry between March 2014 and November 2017, WMLs were graded for 3046 patients and categorized as none (n=1855; 61%), mild (n=608; 20%), or moderate to severe (n=588; 19%). Favorable outcome (modified Rankin Scale score, 0–2) was achieved in 838 patients (49%) without WML, 192 patients (34%) with mild WML, and 130 patients (24%) with moderate-to-severe WML. Increasing WML grades were associated with a shift toward poorer functional outcome in a dose-dependent manner (adjusted common odds ratio, 1.34 [95% CI, 1.13–1.60] for mild WML and 1.67 [95% CI, 1.39–2.01] for moderate-to-severe WML; *P*_trend_, <0.001). Increasing WML grades were associated with futile recanalization (*P*_trend_, <0.001) and were inversely associated with early neurological recovery (*P*_trend_, 0.041) but not with the probability of successful reperfusion or symptomatic intracranial hemorrhage.

**Conclusions:**

An increasing burden of WML at baseline is associated with poorer clinical outcomes after endovascular treatment for acute ischemic stroke but not with the probability of successful reperfusion or symptomatic intracranial hemorrhage.

## Abstract 2

Supplemental Digital Content is available in the text.

Endovascular treatment (EVT) has emerged as the standard of care for patients with acute ischemic stroke due to large vessel occlusion.^1^ Nonetheless, a substantial number of patients treated with EVT are functionally dependent 90 days after stroke, despite successful revascularization. A new area of research has focused on rapid and effective selection of patients who will likely derive most benefit from EVT based on clinical and radiological characteristics. The prognostic significance of certain imaging features, which may reflect either stroke evolution or cerebral vulnerability and resilience, is increasingly recognized.^2,3^ Accordingly, the selection of patients for EVT may shift from a largely time-based approach toward an imaging-based approach.^3^

Many patients with large vessel occlusion concurrently have varying degrees of cerebral small vessel disease, reflected by, among other signs, white matter lesions (WMLs) on baseline neuroimaging.^4^ Cerebral small vessel disease may limit the ability to recover from ischemic stroke^5,6^ or intracerebral hemorrhage.^7^ A higher burden of WML has also been associated with symptomatic intracranial hemorrhage (sICH) after treatment with intravenous thrombolysis.^8,9^ The prognostic importance of WML in patients with ischemic stroke caused by large vessel occlusion and treated with EVT is less well studied. Furthermore, findings from the few studies that assessed WML in the context of EVT have been inconsistent, possibly resulting from the use of selected study populations and varying imaging methods.^10–13^ Information on WML burden is readily available from noncontrast computed tomography (NCCT)^14^ and, when combined with clinical information on other prognostic factors, may inform treatment decisions or become implemented in prognostic models on EVT outcomes.

We aimed to clarify the associations between WML burden, assessed on baseline NCCT, and radiological and clinical outcomes in a large prospective cohort of patients treated with EVT in routine clinical practice in the Netherlands.

## Methods

### Study Protocol, Patient Population, and Clinical Variables

The MR CLEAN Registry (Multicenter Randomized Controlled Trial of Endovascular Treatment for Acute Ischaemic Stroke) is a prospective, multicenter, observational study of patients with acute ischemic stroke treated with EVT in routine clinical practice in the Netherlands in 17 intervention centers.^15^ The current study is based on data from patients included in the MR CLEAN Registry between March 16, 2014, and November 1, 2017. Inclusion criteria for the current analysis were as follows: age ≥18 years; clinical diagnosis of acute ischemic stroke with a proximal arterial occlusion in the anterior circulation (internal carotid artery, middle cerebral artery [M1 and M2], or anterior cerebral artery [A1 and A2]), demonstrated with computed tomography angiography, magnetic resonance angiography, or digital subtraction angiography; and EVT initiated within 6.5 hours of symptom onset or last seen well. Trained study investigators collected clinical and demographic data through chart abstraction from electronic patient records. Local study investigators assessed modified Rankin Scale (mRS)^16^ scores at 90 days (±14 days), as part of routine care.

### Ethics Approval and Data Availability

The study protocol of the MR CLEAN Registry was reviewed by the Erasmus University Medical Center Ethics Committee, which served as the central review board for all participating centers. The requirement for written informed consent was waived, but patients or their representatives were provided with information on the study orally and in writing and were given the opportunity to refuse participation. Source data will not be made available, since no patient approval was obtained for sharing anonymized data. However, detailed analytic methods and study materials, including output files of statistical analyses, will be made available to other researchers on request to the corresponding author.

### Neuroimaging Analysis

An imaging core laboratory, consisting of trained neuroradiologists and interventionists, blinded to all clinical findings except the side of symptoms, determined, among others, the extent of WML and collateral status on baseline (pretreatment) NCCT and single-phase computed tomography angiography, respectively. Pre- and post-EVT extended Thrombolysis in Cerebral Infarction scores were determined on digital subtraction angiography. Before imaging assessments, observers of the imaging core laboratory were given a training set including relevant definitions. The number of individual readers grading each imaging modality varied from 11 to 23. Each scan was graded by a single reader. Hence, no interrater agreement was determined for the imaging variables.

#### Assessment of WMLs

WMLs were graded using a simplified version of the Fazekas scale, which was originally designed for assessment of white matter hyperintensities on magnetic resonance imaging^17^ but has been applied to NCCT previously.^14,18^ In the present study, a total score ranging from 0 to 3 was given to indicate the extent of WMLs in periventricular and deep brain regions combined, depending on their size and confluence (Figure I in the Data Supplement). The score used for statistical analyses was categorized as absent (score 0), mild (score 1), or moderate to severe (score 2 or 3).

#### Assessment of Collaterals

The collateral score was determined with a 4-point visual grading scale,^19^ with a score of 0 for absent collaterals (0% filling of the vascular territory downstream of the occlusion), 1 for poor collaterals (>0% and ≤50% filling), 2 for moderate collaterals (>50% and <100% filling), and 3 for excellent collaterals (100% filling). Interobserver agreement for this method has previously been determined in the MR CLEAN trial and was moderate (κ, 0.60).^20^

#### Assessment of Reperfusion Status

The reperfusion status post-EVT was assessed with the extended Thrombolysis in Cerebral Infarction score, which ranges from 0 (no antegrade reperfusion of the occluded vascular territory) to 3 (complete antegrade reperfusion of the occluded vascular territory).^21^ A score of 2b or higher, which was considered successful reperfusion, could only be given if complete digital subtraction angiography runs including anteroposterior and lateral views were available. If a lateral view was missing, 2a was the highest possible score.

### Outcome Measures

The primary outcome measure was functional outcome, defined as the score on the mRS at 90 days. Secondary clinical outcome measures included mortality at 90 days; futile recanalization, defined as functional dependence (mRS score, ≥3) at 90 days despite successful reperfusion at the end of EVT; and early neurological recovery, defined as a National Institutes of Health Stroke Scale (NIHSS)^22^ score of 0 or 1 at 24 hours after symptom onset or an improvement in NIHSS at 24 hours of at least 8 points compared with baseline NIHSS. Secondary radiological outcome measures were as follows: successful reperfusion at the end of EVT; occurrence of sICH, defined as an ICH that resulted in death or a decline of at least 4 points on the NIHSS, according to the Heidelberg Bleeding Classification^23^; and collateral recruitment at baseline imaging.

### Statistical Analysis

Baseline characteristics of the study population, categorized based on the degree of WML, were compared with standard statistics. We performed ordinal logistic regression to assess the association of WMLs with functional outcome and with collateral recruitment at baseline and binary logistic regression to assess the association of WMLs with futile recanalization, early neurological recovery, successful reperfusion, and occurrence of sICH. In the analysis for successful reperfusion, we performed a post hoc sensitivity analysis excluding patients for whom no 2-dimensional DSA view was available to assess extended Thrombolysis in Cerebral Infarction score after EVT (n=375). For collateral status, the proportional odds assumption was tested and not violated. In all analyses, WMLs were considered an ordinal variable, categorized as absent (score 0), mild (score 1), or moderate to severe (score 2 or 3). Absence of WMLs was used as a reference category in all analyses. Unadjusted and adjusted common odds ratios (ORs) were calculated for each WML category compared with the reference category. Additionally, to assess the presence of a dose-response relationship between exposure (WML) and outcomes, we determined *P*_trend_ in all multivariable analyses.

Prespecified covariates, identified using causal directed acyclic graphs (Figure II in the Data Supplement),^24^ were included in the multivariable models to reduce confounding bias. The analyses for functional outcome, mortality, early neurological recovery, and futile recanalization were adjusted for history of hypertension, age, history of atrial fibrillation, previous stroke, and history of diabetes. The analysis for collaterals was adjusted for age, history of hypertension, and history of diabetes; the analysis for sICH was adjusted for history of hypertension, baseline glucose, and antiplatelet of coumarin use; the analysis for successful reperfusion was adjusted for age, history of hypertension, and baseline glucose. To ascertain that the appropriate confounders had been selected, potential confounders were also selected using a backward elimination procedure, and the effect estimates were compared. In the analysis for functional outcome, we tested for statistical interaction of WML with successful reperfusion and time to reperfusion. In the analysis for sICH, we tested for statistical interaction between WML and intravenous thrombolysis with alteplase.

Multiple imputation was used to impute missing values, including missing WML and outcome variables, using the AregImpute function in R software. All variables used as covariates in the multivariable models were ≥98% complete, except baseline glucose (88.5% complete). As for the outcome variables, mRS at 90 days was known for 93.3% of patients, baseline collateral status for 93.6%, post-EVT extended Thrombolysis in Cerebral Infarction score for 97.4%, delta NIHSS (used for the calculation of early neurological recovery) for 89.3%, and occurrence of sICH for 100%.

Multivariable models were based on imputed values. Sensitivity analyses were performed for each outcome variable using a subset of patients with known values (ie, not imputed) for both WML and the outcome of interest.

All statistical analyses and data visualizations were performed with R software (version 3.6.1; R Foundation).

## Results

### Patient Characteristics and Data Completeness

**Figure 1.**
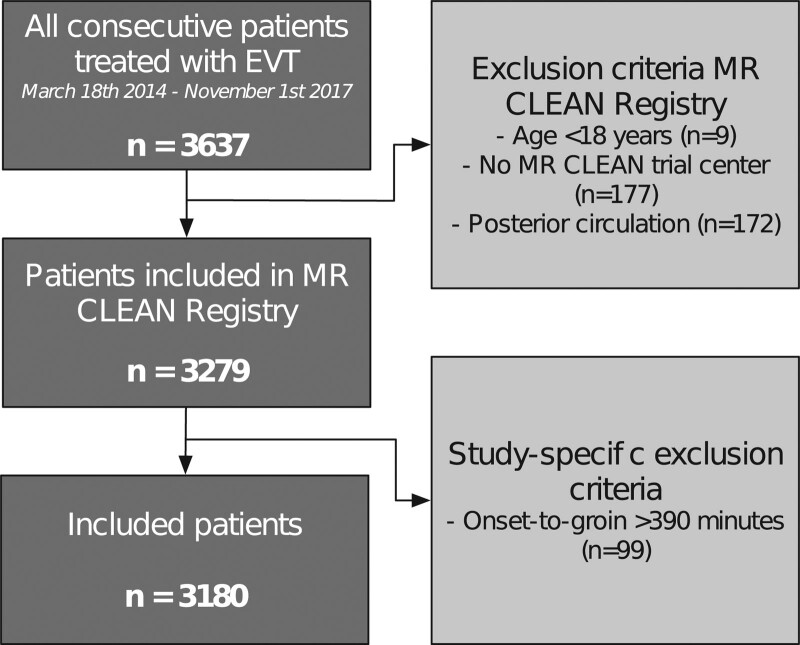
**Flowchart of inclusion of patients in the study.** EVT indicates endovascular treatment; and MR CLEAN, Multicenter Randomized Controlled Trial of Endovascular Treatment for Acute Ischaemic Stroke in the Netherlands.

A total of 3180 patients (median age, 72 years [interquartile range, 61–81]; 1654 [52.0%] men) was included in this analysis (Figure 1). The extent of WML was known for 3046 of 3180 patients (95.8% complete), of whom 1850 (60.7%) had no WML, 608 (20.0%) had mild WML, and 588 (19.7%) had moderate or severe WML. A higher degree of WML was associated with higher age, male sex, and several comorbidities, as well as with a higher NIHSS score, systolic blood pressure, and blood glucose level on admission and longer time from symptom onset to groin puncture (Table I in the Data Supplement). The number of patients with prestroke functional dependency (mRS score of ≥3) was 148 (8.1%) for patients without WML, 85 (14.4%) for mild WML, and 117 (20.5%) for moderate-to-severe WML.

### Primary Outcome

**Figure 2.**
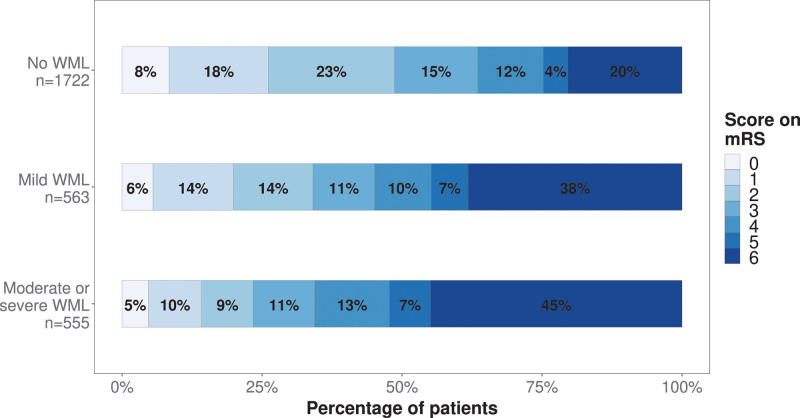
Distribution of 90-d modified Rankin Scale (mRS) scores according to the degree of white matter lesions (WMLs).

**Figure 3.**
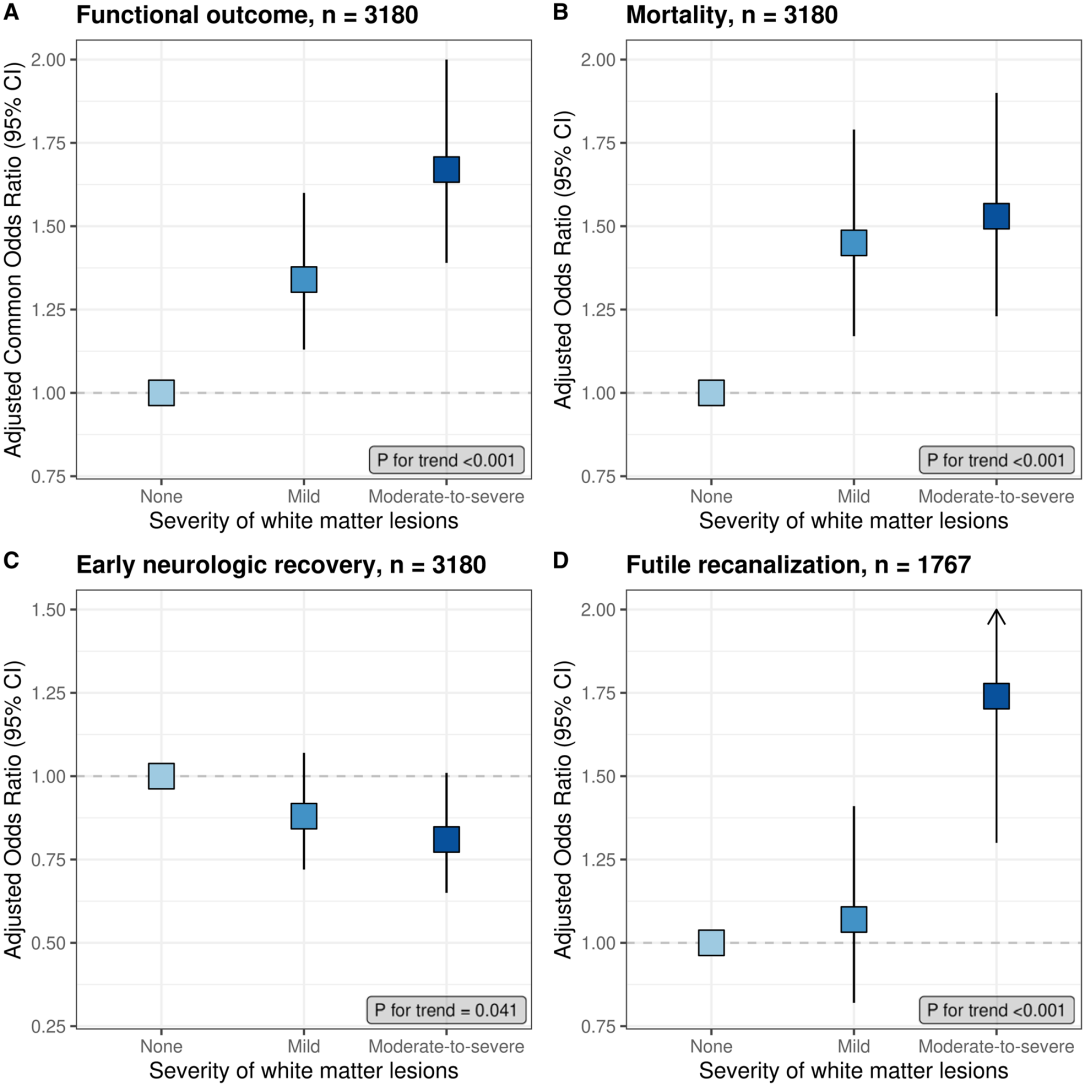
**Associations of white matter lesions with outcomes.** Adjusted associations of increasing degree of white matter lesions (WMLs) with functional outcome (**A**), mortality (**B**), early neurological recovery (**C**), and futile recanalization (**D**). Squares indicate adjusted (common) odds ratio; vertical lines indicate 95% CI. As the absence of WML is the reference category in all models, the adjusted (common) odds ratio is 1.00.

The distribution of mRS scores by WML grade is shown in Figure 2. Favorable outcome (mRS score, 0–2) was achieved in 838 patients (49%) without WML, 192 patients (34%) with mild WML, and 130 patients (24%) with moderate-to-severe WML. Univariable analysis demonstrated a dose-dependent association between the severity of WML and functional outcome (OR, 1.84 [95% CI, 1.55–2.17] for mild WML and 2.79 [95% CI, 2.34–3.33] for moderate-to-severe WML), which persisted in the multivariable model (adjusted common OR, 1.34 [95% CI, 1.13–1.60] for mild WML and 1.67 [95% CI, 1.39–2.01] for moderate-to-severe WML; Table; Figure 3). The sensitivity analysis, including only patients with known WML and mRS score (n=2840), showed largely similar associations (Table). There was no statistically significant interaction between WML and successful reperfusion (*P*=0.47) or time to reperfusion (*P*=0.09) in the analysis for functional outcome.

**Table.**
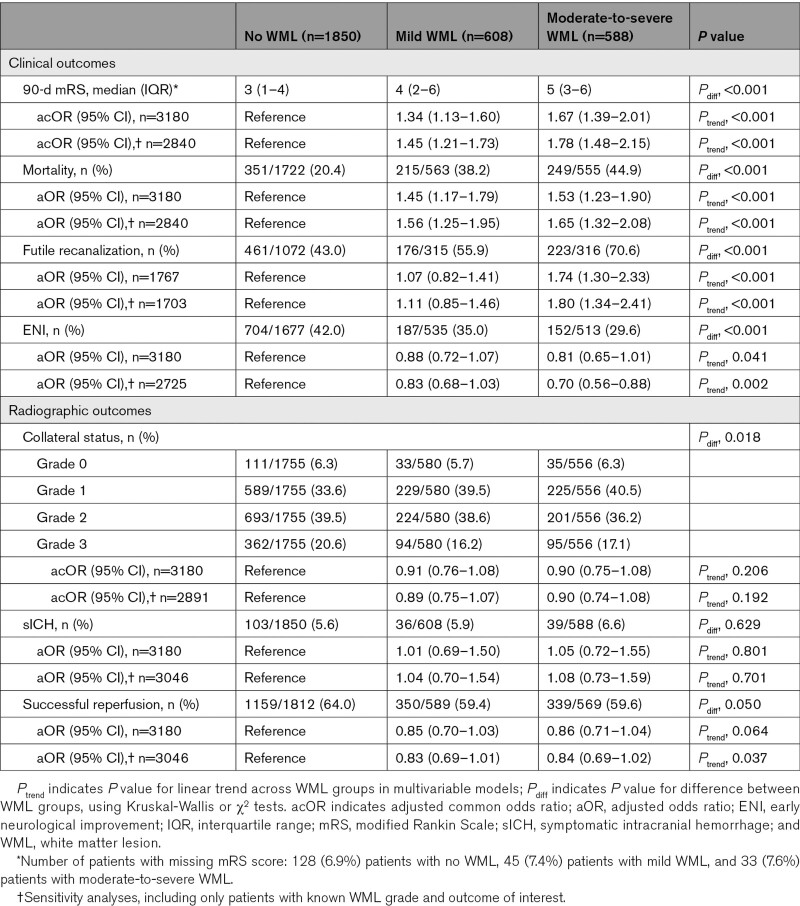
Outcomes and Associations According to the Degree of WMLs

### Secondary Clinical Outcomes

At 90 days, 351 patients (20.4%) without WML had died, as compared with 215 patients (38.2%) with mild WML and 249 patients (44.9%) with moderate-to-severe WML (Table). In unadjusted and adjusted analyses, a dose-dependent increase in the odds of death was observed with increasing WML grades (Table; Figure 3), with similar results in the sensitivity analysis (Table).

Of 2970 patients with known reperfusion status after EVT, 1848 (62.2%) had successful reperfusion. Of the 1767 patients of whom an mRS score at 90 days was also available, 900 (50.9%) had futile recanalization. This proportion increased with higher WML grades (461 patients [43.0%] without WML, 215 patients [55.9%] with mild WML, and 249 patients [70.6%] with moderate-to-severe WML). In univariable analysis, both mild and moderate-to-severe WMLs were associated with futile recanalization (OR, 1.60 [95% CI, 1.25–2.06] and 3.04 [95% CI, 2.32–3.98], respectively). In multivariable analysis, only moderate-to-severe WML was associated with futile recanalization, and this was similar in the sensitivity analysis (Table; Figure 3).

Early neurological recovery was observed in 1071 of 2840 patients (37.8%) with available data and in 704 patients (42.0%) without WML, 187 patients (35.0%) with mild WML, and 152 patients (29.6%) with moderate-to-severe WML. In multivariable analysis, both WML grades were not significantly associated with the probability of early neurological recovery as the upper boundary of the 95% CI crossed 1.0 (OR, 0.88 [95% CI, 0.72–1.07] for mild WML and 0.81 [95% CI, 0.65–1.01] for moderate-to-severe WML). However, there was a linear trend toward a lower probability of early neurological recovery with increasing WML (*P*_trend_, 0.041; Table; Figure 3). In the sensitivity analysis, the association was statistically significant for moderate-to-severe WML (Table).

### Secondary Radiological Outcomes

In univariable analysis, both mild and moderate-to-severe WMLs were associated with worse collaterals at baseline (common OR, 0.83 [95% CI, 0.70–0.98] and 0.79 [95% CI, 0.67–0.94], respectively), but these associations became nonsignificant in the multivariable model (Table). There was no difference across WML grades in occurrence of sICH or successful reperfusion (Table). Likewise, in univariable and multivariable models, WML grade was not associated with the probability of sICH or successful reperfusion (Table). For the post hoc sensitivity analysis including only patients with a 2-dimensional DSA view available (n=2805), the results were similar (data not shown).

Sensitivity analyses using complete WML and outcome data demonstrated largely similar associations (Table). There was no statistical interaction between WML and treatment with intravenous alteplase (*P*=0.52) in the model for sICH.

In all multivariable analyses, when confounders were selected using backward elimination rather than with directed acyclic graphs, effect estimates for WML grades were similar (Table II in the Data Supplement).

## Discussion

In this prospective multicenter cohort study of 3180 patients with acute ischemic stroke treated with EVT, an increasing burden of WML at baseline was independently associated with a lower probability of early neurological recovery, poorer functional outcome and an increased risk of death at 90 days, and futile recanalization. We found no independent association of WML with the occurrence of sICH or probability of successful reperfusion, nor with baseline collateral status.

Preexisting WMLs have been associated with poorer functional outcomes after ischemic stroke in several previous studies.^5,6^ The presumed underlying biological mechanisms are multifold and may involve reduced cerebral network efficiency,^25^ as well as diminished ischemic resilience of the affected tissue.^26,27^ In addition, WMLs are a known risk factor for poststroke depression,^28^ cognitive impairment,^5,29^ and ischemic stroke recurrence,^5,30^ which may all contribute to a limited overall recovery potential. Just about one-quarter of the patients with moderate-to-severe WML in our study reached functional independence 90 days poststroke—a finding comparable to 3 recent reports that studied WML, using either baseline NCCT^10,31^ or magnetic resonance imaging,^11^ in the context of EVT. Similar to our study, WML was an independent predictor of poorer functional outcome in these studies, which strongly suggests that patients with substantial WML have an inherent biological vulnerability to impaired recovery after ischemic stroke. Despite the poorer outcomes of patients with a higher burden of WML, these patients still appear to benefit from EVT, as a recent analysis of the MR CLEAN trial found no effect modification of EVT benefit by WML.^32^ We, therefore, do not recommend taking WML into account in treatment decisions for EVT. Nevertheless, information on WML could help to set realistic recovery expectations after EVT.

In our study, the relation between WML and clinical outcome was already evident at 24 hours, with a lower proportion of patients with moderate-to-severe WML experiencing early neurological recovery. This finding is in line with a recent retrospective analysis of 273 patients^33^ but contrasts with a prospective American study of 389 patients^10^ treated with mechanical thrombectomy. In the latter study, early neurological recovery was achieved in 45% and 44% of patients with none-to-mild WML and moderate-to-severe WML, respectively.^10^ In our cohort, the overall rate of early neurological recovery was lower (38%) and as low as 30% in patients with moderate-to-severe WML. In contrast with our cohort, the American study excluded patients with preexisting functional disability, which could account for the observed differences in early recovery.

In line with previous reports, we found that the relationship of WML with functional outcome was independent of whether successful reperfusion was achieved.^10,11,34,35^ Furthermore, we showed that the relationship of WML with functional outcome was independent of time from symptom onset to reperfusion. Hypothetically, severe WML may shorten the onset-to-reperfusion window for a favorable outcome through faster conversion of salvageable penumbra to infarct core.^27^ WML indeed modified the association between time to reperfusion and functional outcome in an American cohort of 144 patients with successful reperfusion after EVT.^36^ We did not find such modification, which suggests that keeping onset-to-reperfusion times as short as possible is similarly beneficial across WML grades. In the American cohort, patients with moderate-to-severe WML had a shorter onset-to-reperfusion time.

WML may be associated with endothelial dysfunction and disruption of the blood-brain barrier, rendering patients vulnerable to bleeding complications.^37^ Previously, WMLs were shown to be associated with hemorrhagic transformation of ischemic tissue and sICH after intravenous thrombolysis with alteplase,^8,9^ raising concerns about the safety of this treatment before EVT in patients with severe WML. sICH occurred in 5.9% of the patients in our cohort of patients treated with EVT, and WMLs were unrelated to this complication, which is in line with previous reports.^10–13^ Moreover, WML burden was not associated with an increase in the odds of sICH in the subset of patients who received intravenous thrombolysis before EVT.

We found no independent relationship between WML grade and collateral recruitment. This suggests that extensive WML and poor collateral recruitment are independent markers of brain frailty, resulting from shared risk factors such as age and history of hypertension. In 3 previous studies of patients treated with EVT, increasing burden of WML was independently associated with poor collateral recruitment at baseline,^38–40^ but 3 other studies showed no such relationship.^12,41,42^ The inconclusive results may be due to differences in imaging modalities or grading scales for both WML and collateral status. Future studies require more refined, rater-independent measurements of these imaging markers.

Several strengths of our study are worth mentioning. The large sample size and detailed dataset allowed us to examine various clinical and radiographic outcomes and reduce the risk of selection bias and confounding. Selection bias was further limited by inclusion of patients with functional disability at baseline. The fact that the included patients were all treated in routine clinical practice, rather than in the context of a trial, improves generalizability. However, we must also address the limitation that we used NCCT, rather than magnetic resonance imaging, to grade WML. Although NCCT may be inferior in terms of measurement precision, and the use of visual grading increases the risk of phenotype misclassifications, NCCT has the important advantage of its wide availability and routine use in the setting of acute stroke. Moreover, substantial agreement between magnetic resonance imaging and NCCT-based visual grading scales for WML has been demonstrated previously.^14,43^ Second, we did not include patients who were treated beyond 6.5 hours after symptom onset, and our results are, therefore, not generalizable to those patients.

In this MR CLEAN Registry substudy, we demonstrate associations of an increasing burden of WML with poorer clinical outcomes but not with risk of sICH or probability of successful reperfusion. In conclusion, patients with moderate-to-severe WML are at higher risk for poor clinical outcomes but may still benefit from EVT.

## Acknowledgments

We would like to thank the MR CLEAN Registry (Multicenter Randomized Controlled Trial of Endovascular Treatment for Acute Ischaemic Stroke in the Netherlands) investigators; a complete list of the MR CLEAN Registry investigators is provided in the Data Supplement. Dr Uniken Venema contributed to the study concept, statistical analysis, interpretation of the results, and drafting of the manuscript. Dr Lingsma contributed to statistical advice and critical revision of the manuscript. Drs Postma, van den Wijngaard, Albert Vos, Bokkers, Hofmeijer, Dippel, and Majoie contributed to the critical revision of the manuscript. Dr van der Worp contributed to the study concept, interpretation of the results, and critical revision of the manuscript.

## Sources of Funding

The MR CLEAN Registry (Multicenter Randomized Controlled Trial of Endovascular Treatment for Acute Ischaemic Stroke in the Netherlands) was partly funded by the Toegepast Wetenschappelijk Instituut voor Neuromodulatie Foundation, Erasmus MC Medical Center, Maastricht University Medical Center, and Academic Medical Center Amsterdam. Dr Uniken Venema performed this work as coordinating investigator of the trial MR ASAP (Multicentre Randomised Trial of Acute Stroke Treatment in the Ambulance With a Nitroglycerin Patch), which is part of the CONTRAST consortium (Collaboration for New Treatments of Acute Stroke) and is funded by the Netherlands Cardiovascular Research Initiative, an initiative of the Dutch Heart Foundation, and by Stryker Corporation.

## Disclosures

Dr van der Worp has received speaker’s fees from Bayer and Boehringer Ingelheim; served as a consultant to Bayer, Boehringer Ingelheim, and LivaNova; and reports grants from Stryker. Dr Postma reports institutional grant from Siemens Healthcare and Bayer. Dr Majoie received funds from the Toegepast Wetenschappelijk Instituut voor Neuromodulatie Foundation (related to this project, paid to institution) and from CardioVasculair Onderzoek Nederland (CVON)/Dutch Heart Foundation, Stryker, European Commission, and Health Evaluation Program Netherlands (unrelated; all paid to institution) and is shareholder of Nico.lab—a company that focuses on the use of artificial intelligence for medical imaging analysis. Dr Dippel reports that Erasmus MC received grants from Dutch Heart Foundation, Dutch Brain Foundation, The Netherlands Organisation for Health Research and Development, Health Holland Top Sector Life Sciences and Health, Stryker European Operations BV, Thrombolytic Science, LLC, Penumbra, Medtronic, and Cerenovus for new trials in acute stroke treatment. The other authors report no conflicts.

## Supplemental Materials

Online Tables I and II

Online Figures I and II

MR CLEAN Registry Investigators–Group Authors

## Supplementary Material


