# Supplementary material for: White Matter Lesions and Outcomes After Endovascular Treatment for Acute Ischemic Stroke: MR CLEAN Registry Results
Source: Stroke. 2021 Jun 3;52(9):2849–57. doi: 10.1161/STROKEAHA.120.033334 (PMC8378429; doi:10.1161/STROKEAHA.120.033334)
Supplement: Supplementary file 1 [file str-52-2849-s001.pdf]

SUPPLEMENTAL MATERIAL

White matter lesions and outcomes after endovascular treatment for acute ischemic stroke: MR CLEAN Registry results.

Simone M. Uniken Venema, MD; Alida A. Postma, MD, PhD; Ido R. van den Wijngaard, MD, PhD; Jan Albert Vos, MD, PhD; Hester F. Lingsma, PhD; Reinoud P.H. Bokkers, MD, PhD; Jeannette Hofmeijer, MD, PhD; Diederik W. J. Dippel, MD, PhD; Charles B. Majoie, MD, PhD; H. Bart van der Worp, MD, PhD; on behalf of the MR CLEAN Registry investigators.

Supplemental Table I: Baseline characteristics according to the degree of white matter lesions.

	No WML (n = 1850)	Mild WML (n = 608)	Moderate-severe WML (n = 588)	P value
**Demographics**				
Age, *median [IQR]*	67.4 [56.6,76.0]	76.2 [68.7, 83.3]	79.8 [72.9, 86.1]	<0.001
Male sex, *n (%)*	1030/1850 (55.7)	305/608 (50.2)	244/588 (41.5)	<0.001
**Medical history**				
Diabetes mellitus, *n (%)*	259/1850 (14.1)	106/605 (17.5)	119/582 (20.4)	0.001
Hypertension, *n (%)*	859/1815 (47.3)	345/598 (57.7)	359/571 (62.9)	<0.001
Atrial fibrillation, *n (%)*	360/1828 (19.7)	174/604 (28.8)	195/573 (34.0)	<0.001
Myocardial infarction, *n (%)*	232/1817 (12.8)	88/597 (14.7)	99/573 (17.3)	0.022
Peripheral artery disease, *n (%)*	17/18176 (9.7)	51/596 (8.6)	55/573 (9.6)	0.709
Ischemic stroke, *n (%)*	254/1834 (13.8)	109/605 (18.0)	141/582 (24.2)	<0.001
Hypercholesterolemia, *n (%)*	535/1776 (30.1)	182/584 (31.2)	183/552 (33.2)	0.400
Pre-stroke mRS score ≤2, *n (%)*	1671/1819 (91.9)	505/590 (85.6)	453/570 (79.5)	<0.001
Pre-stroke mRS score, *n (%)*				<0.001
0	1341 (73.7)	374 (63.4)	305 (53.5)	
1	216 (11.9)	79 (13.4)	91 (16.0)	
2	114 (6.3)	52 (8.8)	57 (10.0)	
3	87 (4.8)	46 (7.8)	67 (11.8)	
4	48 (2.6)	34 (5.8)	43 (7.5)	
5	13 (0.7)	5 (0.8)	7 (1.2)	
**Medications and intoxications**				
Statin, *n (%)*	596/1822 (32.7)	213/590 (36.1)	250/570 (43.9)	<0.001
Antihypertensive, *n (%)*	881/1822(48.4)	348/593(58.7)	392/577 (67.9)	<0.001
Antiplatelet, *n (%)*	529/1832 (28.9)	195/601 (32.4)	217/578 (37.5)	<0.001
Vitamin K antagonist, *n (%)*	197/1839 (10.7)	82/602 (13.6)	108/584 (18.5)	<0.001
DOAC, *n (%)*	5/18349 (3.2)	20/597 (3.4)	20/581 (3.4)	0.961
Current smoking, *n (%)*	459/1470 (31.2)	105/448 (23.4)	92/427 (21.5)	<0.001
**Admission variables and baseline imaging**				
NIHSS, *median [IQR]*	15 [11, 19]	16 [11, 19]	17 [12, 20]	0.003
Systolic blood pressure, *mean (SD)*	148 (24)	152 (25)	155 (26)	<0.001
Baseline glucose, *median [IQR]*	6.7 [5.9, 8.0]	6.7 [6.0, 8.0]	7.0 [6.1, 8.4]	0.006
Left hemisphere, *n (%)*	976/1850 (52.8)	326/608 (53.6)	308/587 (52.5)	0.935
Occluded segment, *n (%)*				0.007
ICA	111/1798 (6.2)	24/588 (4.1)	14/570 (2.5)	
ICA-T	367/1798 (20.4)	131/588 (22.3)	119/570 (20.9)	
M1	1041/1798 (57.9)	341/588 (58.0)	335/570 (58.8)	
M2	265/1798 (14.7)	85/588 (14.5)	101/570 (17.7)	
Other (M3, A1, A2)	14/1798 (0.8)	7/588 (1.2)	1/570 (0.2)	
ASPECTS, *median (IQR)*	9 [8, 10]	9 [7, 10]	9 [8, 10]	0.351
Ipsilateral carotid stenosis, *n (%)*				0.131
<50% stenosis at bifurcation	1324/1640 (80.7)	440/546 (80.6)	421/533 (79.0)	
≥50% stenosis at bifurcation	128/1640 (7.8)	50/546 (9.2)	60/533 (11.3)	
Occlusion at bifurcation	188/1640 (11.5)	56/546 (10.3)	52/533 (9.8)	
**Workflow and treatments**				
IV alteplase before EVT, *n (%)*	1435/1843 (77.9)	459/608 (75.5)	434/585 (74.2)	0.139
Time from onset to baseline NCCT (minutes), *median (IQR)*	70 [51, 111]	72 [52, 110]	73 [54, 125]	0.262
Time from onset to groin puncture (minutes), *median (IQR)*	190 [147, 250]	190 [150, 243]	202 [160, 260]	0.001
Time from onset to successful reperfusion or last contrast bolus (minutes), *median (IQR)*	248 [195, 310]	245 [200, 310]	258 [208, 314]	0.086
General anesthesia, *n (%)*	428/1753 (24.4)	158/561 (28.2)	139/545 (25.5)	0.044

WML indicates white matter lesions; mRS, modified Rankin Scale score; DOAC, direct oral anticoagulant; NIHSS, National Institutes of Health Stroke Scale; ICA(-T), intracranial carotid artery (terminus); ASPECTS, Alberta Stroke Program Early CT Score; IV intravenous ; NCCT, non-contrast computed tomography.

Number of missing values in non-categorical variables for no WML, mild WML and moderate-severe WML, respectively: NIHSS (31, 11, 6), systolic blood pressure (49, 17, 18), baseline glucose (199, 72, 73), ASPECTS (3, 1, 2); time from symptom onset to baseline NCCT (503, 183, 175), groin puncture (10, 1, 2) and reperfusion (97, 43, 52).

Supplemental Table II: Overview of variables adjusted for in the multivariable analyses when confounders were selected using DAGs or backward selection, and corresponding effect estimates for the relationship between WML and each outcome.

Outcome	*Confounders selected with DAG*	*Adjusted (common) odds ratio [95% CI] with confounders selected with DAG*	*Confounders selected with backward selection*	*Adjusted (common) odds ratio [95% CI] with confounders selected with backward regression*
90-day mRS score	Age, history of hypertension, history of atrial fibrillation, previous stroke and history of diabetes mellitus	Mild WML = 1.34 [1.13-1.60]<br>Moderate-to-severe WML = 1.67 [1.39-2.01]	Age, time from symptom onset to groin puncture, time from symptom onset to reperfusion, baseline glucose, systolic blood pressure, baseline NIHSS, collateral status, baseline ASPECTS, history of diabetes mellitus, pre-stroke mRS, use of statins, use of coumarins, use of antiplatelets	Mild WML = 1.39 [1.16-1.66]<br>Moderate-to-severe WML = 1.69 [1.38-2.07]
90-day mortality	Age, history of hypertension, history of atrial fibrillation, previous stroke and history of diabetes mellitus	Mild WML = 1.45 [1.17-1.79] <0.001<br>Moderate-to-severe WML = 1.53 [1.23-1.90] <0.001	Age, time from symptom onset to groin puncture, time from symptom onset to reperfusion, baseline glucose, systolic blood pressure, baseline NIHSS, collateral status, baseline ASPECTS, history of diabetes mellitus, pre-stroke mRS, use of statins, use of coumarins, use of antiplatelets	Mild WML = 1.51 [1.20-1.91]<br>Moderate-to-severe WML = 1.46 [1.15-1.86]
Futile recanalization	Age, history of hypertension, history of atrial fibrillation, previous stroke and history of diabetes mellitus	Mild WML = 1.07 (0.82-1.41)<br>Moderate-to-severe WML = 1.74 (1.30-2.33)	Age, sex, use of coumarins, use of statins, previous stroke, history of diabetes mellitus, collateral status, baseline NIHSS, baseline glucose, duration from symptom onset to reperfusion, duration from symptom onset to CTA	Mild WML = 1.19 [0.80-1.77]<br>Moderate-to-severe WML = 1.68 [1.11-2.53]
Early neurologic improvement	Age, history of hypertension, history of atrial fibrillation, previous stroke and history of diabetes mellitus	Mild WML = 0.88 [0.72-1.07]<br>Moderate-to-severe WML = 0.81 [0.65-1.01]	Age, pre-stroke mRS, previous stroke, baseline ASPECTS, collateral status, baseline NIHSS, systolic blood pressure, baseline glucose, time to groin puncture, time from symptom onset to reperfusion	Mild WML = 0.88 [0.71-1.09]<br>Moderate-to-severe WML = 0.83 [0.66-1.04]
Collateral status	Age, history of hypertension, history of diabetes mellitus	Mild WML = 0.91 [0.76-1.08]<br>Moderate-to-severe WML = 0.90 [0.75-1.08]	Age, sex, history of peripheral artery disease, baseline NIHSS, occluded segment, baseline ASPECTS, extracranial internal carotid artery disease	Mild WML = 0.94 [0.79-1.13]<br>Moderate-to-severe WML = 0.93<br>[0.77-1.13]
sICH	History of hypertension, baseline glucose, use of antiplatelets or vitamin K antagonists	Mild WML = 1.01 [0.69-1.50]<br>Moderate-to-severe WML = 1.05 [0.72-1.55]	Baseline glucose, systolic blood pressure, collaterals, use of antiplatelets	Mild WML = 1.00 [0.67-1.47]<br>Moderate-to-severe WML = 1.01 [0.69-1.49]
Successful reperfusion	History of hypertension, baseline glucose	Mild WML = 0.85 [0.70-1.03]<br>Moderate-to-severe WML = 0.86 [0.71-1.04]	History of atrial fibrillation, systolic blood pressure, time from onset to reperfusion, general anesthesia	Mild WML = 0.83 [0.69-1.01]<br>Moderate-to-severe WML = 0.91 [0.75-1.11]

Futile recanalization was defined as functional dependence (mRS $\geq$ 3) at 90 days despite successful reperfusion at the end of EVT (an eTICI score of 2B or higher). Early neurologic recovery was defined as a NIHSS score of 0 or 1 at 24 hours after symptom onset, or an improvement in NIHSS at 24 hours of at least 8 points compared to baseline NIHSS. sICH was defined as an ICH that resulted in death or a decline of at least 4 points on the NIHSS, according to the Heidelberg Bleeding Classification.
DAG, directed acyclic graph; WML, white matter lesions; mRS, modified Rankin Scale; (s)ICH, (symptomatic) intracranial hemorrhage; NIHSS, national Institutes of Health Stroke Scale; CTA, computed tomography angiography; ASPECTS, Alberta stroke program early CT score.

Supplemental Figure I: Grading scale used to grade white matter lesions.

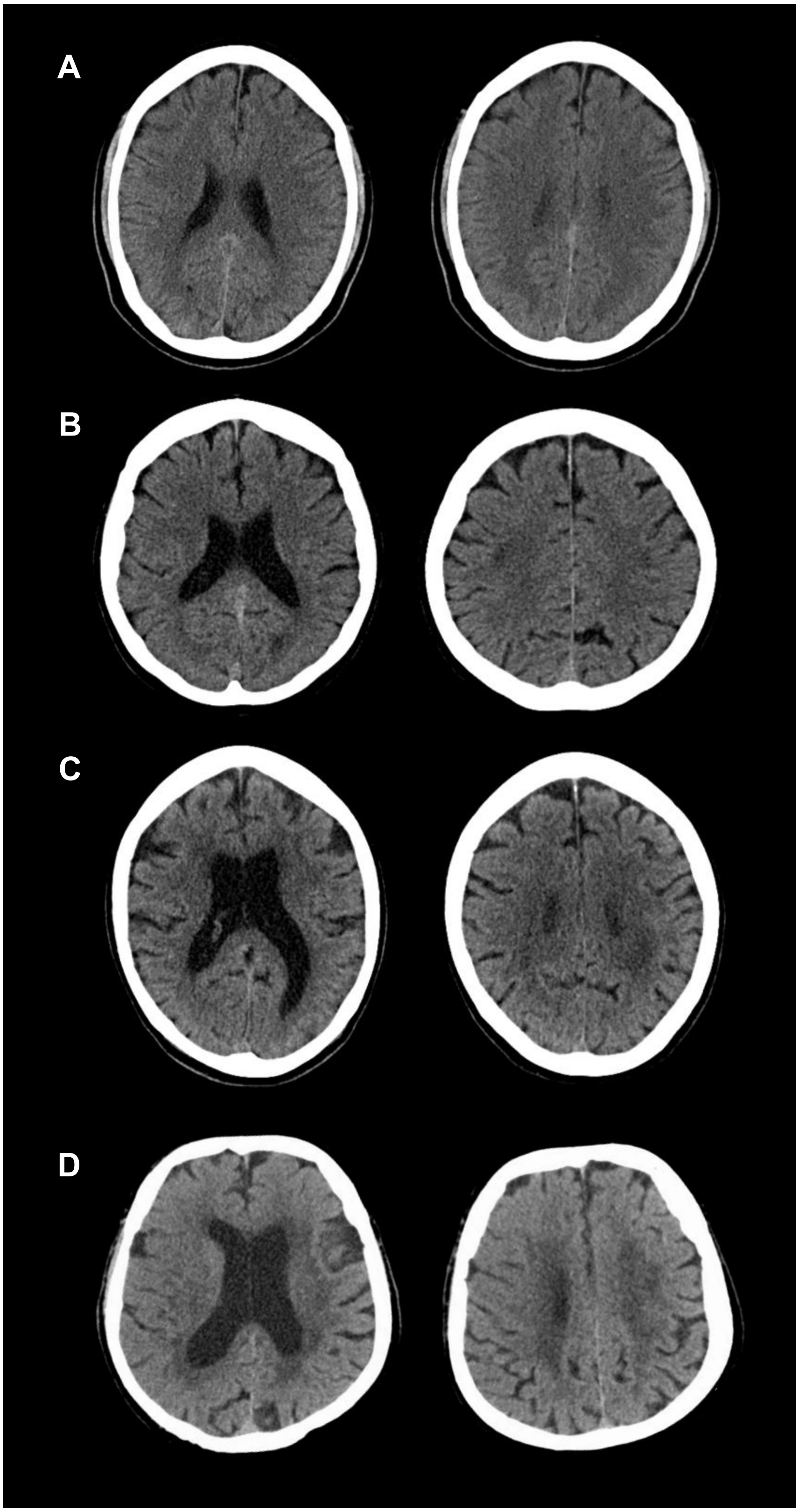

Radiologists from the imaging core lab assessed the total burden of WML. A: no WML (grade 0; absence of periventricular or deep WML), B: mild WML (grade 1; capping of the ventricles or punctate focal deep WML), C: moderate WML (grade 2; more extensive periventricular WML or early confluence of focal deep WML), D: severe WML (grade 3; confluent, irregular periventricular WMH extending to the cortex, or large confluent areas of deep WML).

Supplemental Figure II: Directed Acyclic Graphs

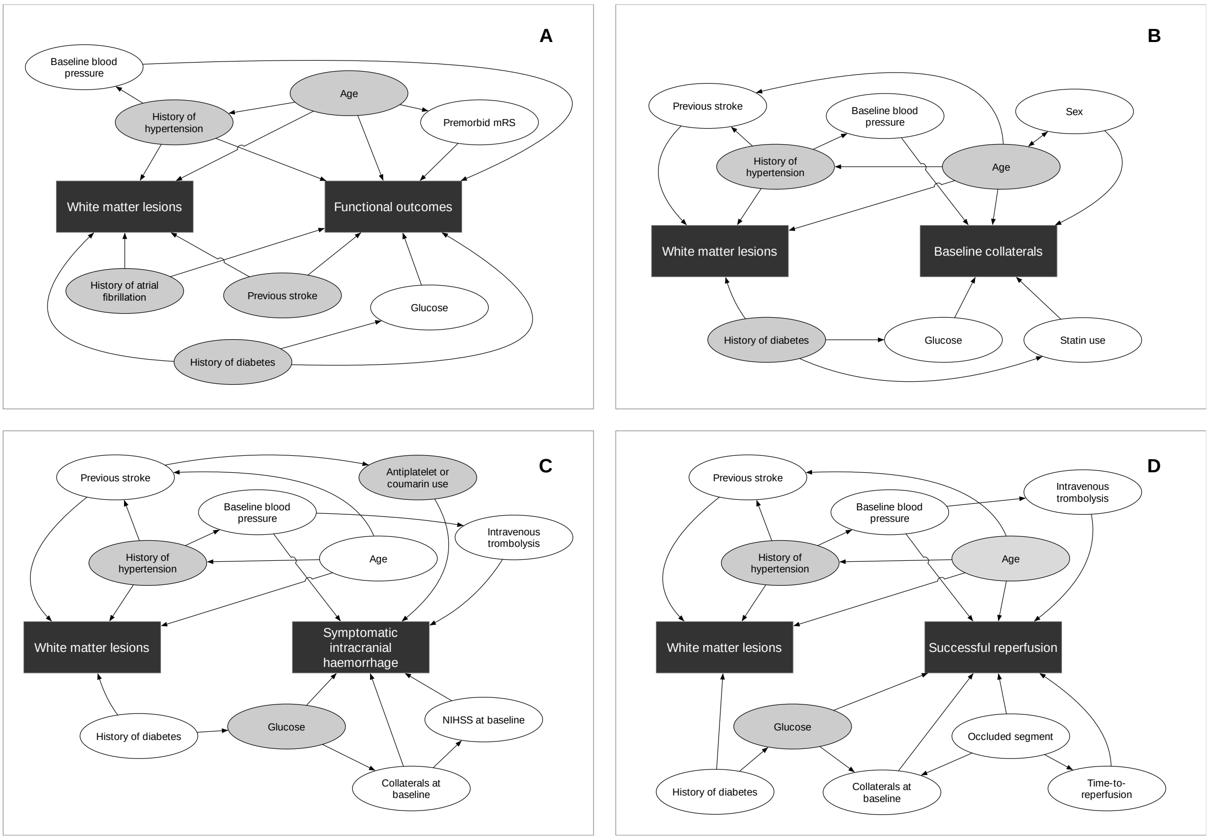

Directed acyclic graphs (DAGs) were used to select potential confounders for the relationship between white matter lesions with each outcome variable. Variables in grey ellipses are potential confounders; once they are removed, there is no direct or indirect path (i.e., via variables in white ellipses) between the predictor variable (white matter lesions) and the outcome variable. In analyses for functional outcome (A), we adjusted for age, history of hypertension, history of atrial fibrillation, previous stroke and history of diabetes mellitus. We used the same potential confounders in analyses for mortality, early neurologic recovery and futile recanalization. In the analysis for baseline collaterals (B), we adjusted for age, history of hypertension and history of diabetes; for symptomatic intracranial hemorrhage (C), we adjusted for history of hypertension, baseline glucose and use of antiplatelets or vitamin K antagonists; for successful reperfusion (D), we adjusted for history of hypertension, baseline glucose and age.

MR CLEAN Registry Investigators – group authors

Executive committee

Diederik W.J. Dippel[1];Aad van der Lugt[2];Charles B.L.M. Majoie[3];Yvo B.W.E.M. Roos[4];Robert J. van Oostenbrugge[5];Wim H. van Zwam[6];Jelis Boiten[14];Jan Albert Vos[8]

Study coordinators

Ivo G.H. Jansen[3];Maxim J.H.L. Mulder[1,2];Robert- Jan B. Goldhoorn[5,6];Kars C.J. Compagne[2];Manon Kappelhof[3];Josje Brouwer[4];Sanne J. den Hartog[1,2,40];Wouter H. Hinsenveld [5,6];

Local principal investigators

Diederik W.J. Dippel[1];Bob Roozenbeek[1];Aad van der Lugt[2];Adriaan C.G.M. van Es[2];Charles B.L.M. Majoie[3];Yvo B.W.E.M. Roos[4];Bart J. Emmer[3];Jonathan M. Coutinho[4];Wouter J. Schonewille[7];Jan Albert Vos[8]; Marieke J.H. Wermer[9];Marianne A.A. van Walderveen[10];Julie Staals[5];Robert J. van Oostenbrugge[5];Wim H. van Zwam[6];Jeannette Hofmeijer[11];Jasper M. Martens[12];Geert J. Lycklama à Nijeholt[13];Jelis Boiten[14];Sebastiaan F. de Bruijn[15];Lukas C. van Dijk[16];H. Bart van der Worp[17];Rob H. Lo[18];Ewoud J. van Dijk[19];Hieronymus D. Boogaarts[20];J. de Vries[22];Paul L.M. de Kort[21]; Julia van Tuijl[21] ; Jo P. Peluso[26];Puck Fransen[22];Jan S.P. van den Berg[22];Boudewijn A.A.M. van Hasselt[23];Leo A.M. Aerden[24];René J. Dallinga[25];Maarten Uyttenboogaart[28];Omid Eschgi[29];Reinoud P.H. Bokkers[29];Tobien H.C.M.L. Schreuder[30];Roel J.J. Heijboer[31];Koos Keizer[32];Lonneke S.F. Yo[33];Heleen M. den Hertog[22];Emiel J.C. Sturm[35];Paul J.A.M. Brouwers[34]

Imaging assessment committee

Charles B.L.M. Majoie[3](chair);Wim H. van Zwam[6];Aad van der Lugt[2];Geert J. Lycklama à Nijeholt[13];Marianne A.A. van Walderveen[10];Marieke E.S. Sprengers[3];Sjoerd F.M. Jenniskens[27];René van den Berg[3];Albert J. Yoo[38];Ludo F.M. Beenen[3];Alida A. Postma[6];Stefan D. Roosendaal[3];Bas F.W. van der Kallen[13];Ido R. van den Wijngaard[13];Adriaan C.G.M. van Es[2];Bart J. Emmer·[3];Jasper M. Martens[12]; Lonneke S.F. Yo[33];Jan Albert Vos[8]; Joost Bot[36], Pieter-Jan van Doormaal[2]; Anton Meijer[27];Elyas Ghariq[13]; Reinoud P.H. Bokkers[29];Marc P. van Proosdij[37];G. Menno Krietemeijer[33];Jo P. Peluso[26];Hieronymus D. Boogaarts[20];Rob Lo[18];Dick Gerrits[35];Wouter Dinkelaar[2]Auke P.A. Appelman[29];Bas Hammer[16];Sjoert Pegge[27];Anouk van der Hoorn[29];Saman Vinke[20].

Writing committee

Diederik W.J. Dippel[1](chair);Aad van der Lugt[2];Charles B.L.M. Majoie[3];Yvo B.W.E.M. Roos[4];Robert J. van Oostenbrugge[5];Wim H. van Zwam[6];Geert J. Lycklama à Nijeholt[13];Jelis Boiten[14];Jan Albert Vos[8];Wouter J. Schonewille[7];Jeannette Hofmeijer[11];Jasper M. Martens[12];H. Bart van der Worp[17];Rob H. Lo[18]

Adverse event committee

Robert J. van Oostenbrugge[5](chair);Jeannette Hofmeijer[11];H. Zwenneke Flach[23]

Trial methodologist

Hester F. Lingsma[40]

Research nurses / local trial coordinators

Naziha el Ghannouti[1];Martin Sterrenberg[1];Wilma Pellikaan[7];Rita Sprengers[4];Marjan Elfrink[11];Michelle Simons[11];Marjolein Vossers[12];Joke de Meris[14];Tamara Vermeulen[14];Annet Geerlings[19];Gina van Vemde[22];Tiny Simons[30];Gert Messchendorp[28];Nynke Nicolaij[28];Hester Bongenaar[32];Karin Bodde[24];Sandra Kleijn[34];Jasmijn Lodico[34]; Hanneke Droste[34];Maureen Wollaert[5];Sabrina Verheesen[5];D. Jeurrissen[5];Erna Bos[9];Yvonne Drabbe[15];Michelle Sandiman[15];Nicoline Aaldering[11];Berber Zweedijk[17];Jocova Vervoort[21];Eva Ponjee[22];Sharon Romviel[19];Karin Kanselaar[19];Denn Barning[10].

PhD / Medical students:

Esmee Venema[40]; Vicky Chalos[1,40]; Ralph R. Geuskens[3]; Tim van Straaten[19];Saliha Ergezen[1]; Roger R.M. Harmsma[1]; Daan Muijres[1]; Anouk de Jong[1];Olvert A. Berkhemer[1,3,6];Anna M.M. Boers[3,39]; J. Huguet[3];P.F.C. Groot[3];Marieke A. Mens[3];Katinka R. van Kranendonk[3];Kilian M. Treurniet[3];Manon L. Tolhuisen[3,39];Heitor Alves[3];Annick J. Weterings[3],Eleonora L.F. Kirkels[3],Eva

J.H.F. Voogd[11];Lieve M. Schupp[3];Sabine L. Collette[28,29];Adrien E.D. Groot[4];Natalie E. LeCouffe[4];Praneeta R. Konduri[39];Haryadi Prasetya[39];Nerea Arrarte-Terreros[39];Lucas A. Ramos[39].

List of affiliations

Department of Neurology[1], Radiology[2], Public Health[40], Erasmus MC University Medical Center;
Department of Radiology and Nuclear Medicine[3], Neurology[4], Biomedical Engineering & Physics[39], Amsterdam UMC, University of Amsterdam, Amsterdam;
Department of Neurology[5], Radiology[6], Maastricht University Medical Center and Cardiovascular Research Institute Maastricht (CARIM);
Department of Neurology[7], Radiology[8], Sint Antonius Hospital, Nieuwegein;
Department of Neurology[9], Radiology[10], Leiden University Medical Center;
Department of Neurology[11], Radiology[12], Rijnstate Hospital, Arnhem;
Department of Radiology[13], Neurology[14], Haaglanden MC, the Hague;
Department of Neurology[15], Radiology[16], HAGA Hospital, the Hague;
Department of Neurology[17], Radiology[18], University Medical Center Utrecht;
Department of Neurology[19], Neurosurgery[20], Radiology[27], Radboud University Medical Center, Nijmegen;
Department of Neurology[21], Radiology[26], Elisabeth-TweeSteden ziekenhuis, Tilburg;
Department of Neurology[22], Radiology[23], Isala Klinieken, Zwolle;
Department of Neurology[24], Radiology[25], Reinier de Graaf Gasthuis, Delft;
Department of Neurology[28], Radiology[29], University Medical Center Groningen;
Department of Neurology[30], Radiology[31], Atrium Medical Center, Heerlen;
Department of Neurology[32], Radiology[33], Catharina Hospital, Eindhoven;
Department of Neurology[34], Radiology[35], Medical Spectrum Twente, Enschede;
Department of Radiology[36], Amsterdam UMC, Vrije Universiteit van Amsterdam, Amsterdam;
Department of Radiology[37], Noordwest Ziekenhuisgroep, Alkmaar;
Department of Radiology[38], Texas Stroke Institute, Texas, United States of America.
