# Supplementary figures and images for: White Matter Lesions and Outcomes After Endovascular Treatment for Acute Ischemic Stroke: MR CLEAN Registry Results
Source: Stroke. 2021 Jun 3;52(9):2849–57. doi: 10.1161/STROKEAHA.120.033334 (PMC8378429; doi:10.1161/STROKEAHA.120.033334)

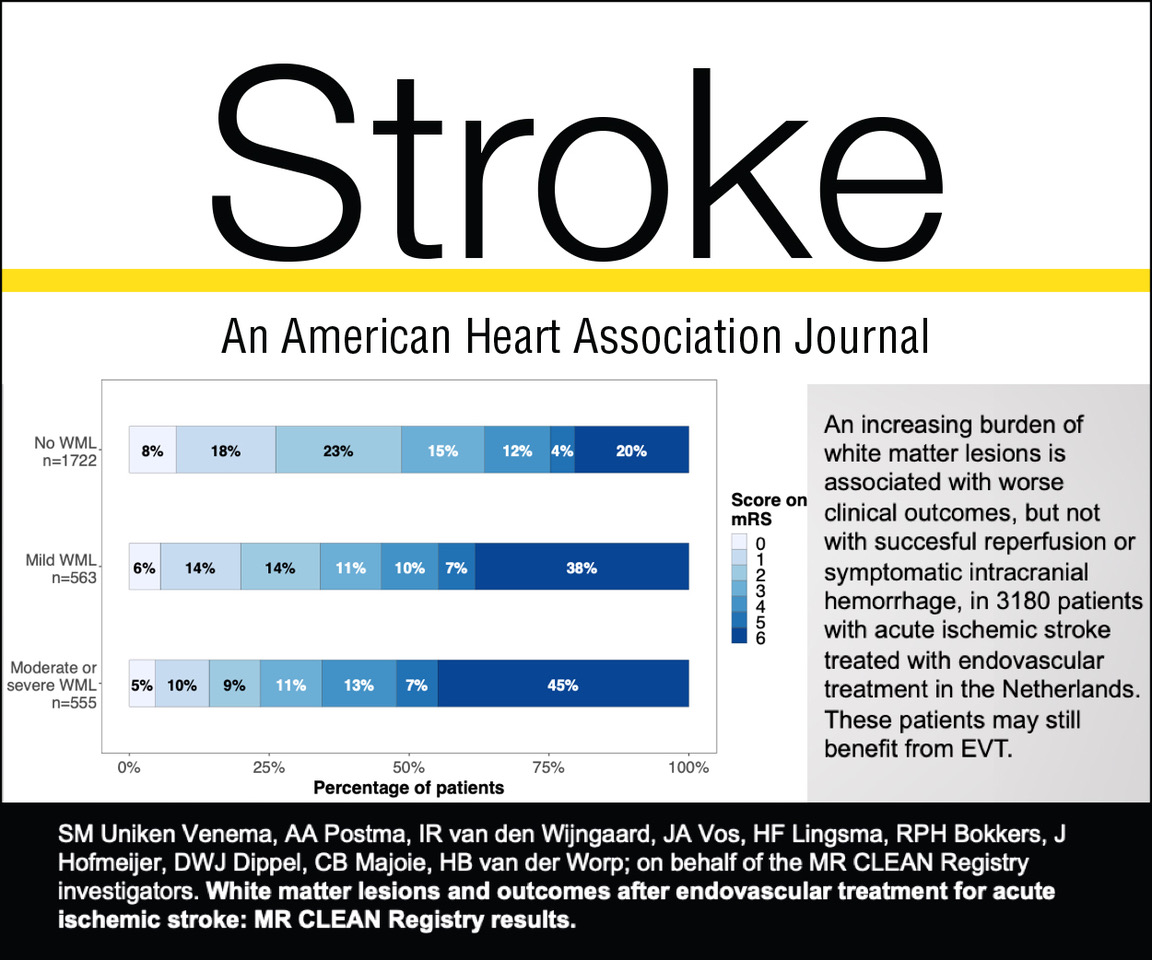

Supplement: Supplementary file 2 [file str-52-2849-s002.jpg]
